# 巨噬细胞胞葬驱动肺癌微环境结构重塑与血管生成的机制及临床干预前景

**DOI:** 10.3779/j.issn.1009-3419.2026.101.08

**Published:** 2026-04-20

**Authors:** Yu QIN, Hongmei YUE, Yongqing WANG, Zhangfan ZHU, Fan YANG

**Affiliations:** ^1^730000 兰州，兰州大学第一临床医学院; ^1^The First Clinical Medical School, Lanzhou University, Lanzhou 730000, China; ^2^730000 兰州，兰州大学第一医院呼吸与危重症医学科; ^2^Department of Respiratory and Critical Care Medicine, The First Hospital of Lanzhou University, Lanzhou 730000, China

**Keywords:** 肺肿瘤, 巨噬细胞, 胞葬, 肿瘤微环境, 代谢重编程, 血管生成, Lung neoplasms, Macrophages, Efferocytosis, Tumor microenvironment, Metabolic reprogramming, Angiogenesis

## Abstract

肺癌是全球发病率和死亡率均居首位的恶性肿瘤。近年来免疫检查点抑制剂显著改善了部分晚期非小细胞肺癌患者的生存预后，但仍有相当比例患者存在原发性或继发性免疫耐药。肿瘤微环境（tumor microenvironment, TME）结构重塑被认为是影响免疫治疗疗效的重要因素。胞葬是肿瘤相关巨噬细胞（tumor-associated macrophages, TAMs）清除凋亡细胞的重要过程，在维持炎症消退和组织稳态的同时，可诱导巨噬细胞发生显著的代谢重编程及分泌谱改变。持续活化的胞葬作用通过调控脂质代谢和糖酵解等代谢途径，促进多种免疫抑制及组织修复因子的分泌，从而驱动异常血管生成、激活肿瘤相关成纤维细胞并促进细胞外基质沉积，导致肿瘤基质结构重塑，形成免疫排斥型TME，限制效应T细胞浸润并降低免疫治疗疗效。本文综述胞葬作用在TME中的分子机制，重点阐述其在代谢重编程、血管生成异常及基质纤维化中的作用，并探讨靶向胞葬相关信号通路及TME结构重塑的潜在治疗策略，并进一步探讨胞葬相关指标在免疫检查点抑制剂疗效预测的潜在价值，以期为肺癌免疫治疗耐药的精准干预提供新的研究思路。

## Abstract (en)

Lung cancer is one of the most prevalent and lethal malignant tumors worldwide. In recent years, immune checkpoint inhibitors have significantly improved the survival outcomes of some patients with advanced non-small cell lung cancer; however, primary and acquired resistance remain important barriers limiting their clinical efficacy. Research has revealed that structural remodeling of the tumor microenvironment (TME) is one of the key factors involved in immunotherapy resistance. Efferocytosis is an important process by which tumor-associated macrophages clear apoptotic cells. In the lung cancer TME, the high apoptotic cell burden can lead to persistent activation of efferocytosis. Studies have shown that sustained efferocytosis is not merely a process of cellular debris clearance, but can also induce metabolic reprogramming in macrophages, including dysregulated lipid metabolism and enhanced glycolysis, and promote the secretion of immunosuppressive and tissue-repair-related factors. These changes further promote pathological angiogenesis, activation of cancer-associated fibroblasts, and excessive extracellular matrix deposition, thereby driving structural remodeling of the TME and forming an immune-excluded microenvironment characterized by vascular abnormalities and stromal fibrosis. This process restricts effector T-cell infiltration and impairs the efficacy of immune checkpoint inhibitors. This review describes the molecular mechanisms of macrophage efferocytosis in the lung cancer TME, focusing on its regulatory roles in metabolic reprogramming, pathological angiogenesis, and stromal fibrosis, and discusses potential therapeutic strategies targeting efferocytosis-related signaling pathways and TME structural remodeling, aiming to provide new insights into overcoming immunotherapy resistance in lung cancer.

肺癌是全球发病率和死亡率均居首位的恶性肿瘤^[1]^，免疫检查点抑制剂（immune checkpoint inhibitors, ICIs）虽显著改善了部分晚期非小细胞肺癌（non-small cell lung cancer, NSCLC）患者的预后^[2]^，仍有许多患者存在原发性或继发性免疫耐药。肿瘤相关巨噬细胞（tumor-associated macrophages, TAMs）是肺癌肿瘤微环境（tumor microenvironment, TME）中发挥核心功能的先天免疫细胞，其介导的胞葬作用^[3]^能够特异性地清除凋亡细胞。肺癌TME中因化疗、放疗、慢性缺氧以及持续炎症存在高负荷凋亡细胞，使胞葬作用处于持续激活状态。胞葬可通过促进异常血管生成、激活肿瘤相关成纤维细胞（cancer-associated fibroblasts, CAFs）、介导细胞外基质（extracellular matrix, ECM）异常沉积，驱动肿瘤基质结构重塑，并可能促进免疫排斥性TME的形成，进而影响免疫治疗疗效。目前，巨噬细胞胞葬调控肺癌TME结构重塑的分子机制尚未得到系统阐释，本文综述肺癌TME中巨噬细胞胞葬作用的分子机制，重点总结其在代谢重编程、血管生成异常及基质纤维化中的核心调控作用，并探讨靶向胞葬相关信号通路的潜在临床干预策略，为肺癌免疫治疗的研究与优化提供新的思路。

## 1 巨噬细胞胞葬引发的代谢重编程及其分泌效应

在肺癌TME中，高负荷的凋亡细胞被TAMs特异性识别并吞噬，使胞葬作用持续激活。TAMs是肺癌TME中数量最丰富的先天免疫细胞，是连接细胞死亡、炎症调控、基质重塑与血管生成的关键枢纽^[4]^。胞葬作用由TAMs受体酪氨酸激酶家族（Tyro3, Axl, MerTK）介导，通过识别磷脂酰丝氨酸（phosphatidylserine, PS）及其桥接分子[如：生长阻滞特异性蛋白6（growth arrest specific 6, Gas6）、蛋白S（protein S, ProS）]激活独特的信号级联反应，进而诱导巨噬细胞发生深度代谢重编程与表型转换^[5]^，形成特征性的胞葬后分泌组，为肿瘤基质结构重塑提供重要的分子基础。

### 1.1 脂质代谢与核受体激活

胞葬过程中，TAMs吞噬凋亡细胞后胞内脂质负荷急剧升高。已有研究^[6]^指出，在肺腺癌组织中，TAMs呈现出不稳定的脂质代谢，胆固醇对巨噬细胞基因表达有明显影响作用。为维持脂质稳态，其通过溶酶体酸性脂肪酶降解脂滴，释放的脂肪酸和氧固醇作为天然配体，激活核受体肝X受体（liver X receptor, LXR）及过氧化物酶体增殖物激活受体-γ（peroxisome proliferator-activated receptor-γ, PPAR-γ）^[7]^。在肺癌TME中，激活的PPAR-γ/LXR信号轴发挥双重调控作用：一方面，LXR靶基因腺苷三磷酸结合盒亚家族A成员1（ATP-binding cassette subfamily A member 1, *ABCA1*）和腺苷三磷酸结合盒亚家族G成员1（ATP-binding cassette subfamily G member 1, *ABCG1*）作为胆固醇外排关键转运体，可避免TAMs因脂质过载转化为泡沫细胞，维持细胞功能稳定性^[8]^；另一方面，该信号轴可诱导TAMs持续高表达MerTK受体及转化生长因子-β（transforming growth factor-β, TGF-β），营造利于肿瘤生长的免疫抑制TME^[9]^。同时，TAMs中积累的脂质可通过ABCA1介导的胆固醇外排途径重新分配，可能为增殖中的肿瘤细胞提供代谢产物，形成一定程度的代谢共生关系^[10]^。研究^[11]^指出，肺腺癌中脂质代谢基因T细胞淋巴瘤侵袭转移蛋白2（T-cell lymphoma invasion and metastasis 2, *TIAM2*）基因的高表达与M2促瘤表型TAMs极化密切相关，提示脂质代谢重编程可能是免疫抑制TME形成的重要驱动因素，这种“脂质-核受体-细胞因子”级联反应，不仅强化TAMs的后续吞噬能力，也可能使其成为持续分泌免疫抑制与组织修复因子的代谢调控节点。

### 1.2 糖酵解增强与乳酸堆积

糖代谢与脂质代谢在TAMs功能编程及肺癌进展中存在紧密的串扰协同，胞葬启动后，TAMs迅速偏离氧化磷酸化表型，通过上调葡萄糖转运体1增强葡萄糖摄取，依赖磷脂酰肌醇3-激酶（phosphoinositide 3-kinase, PI3K）/蛋白激酶B（protein kinase B, AKT）信号通路优先启动有氧糖酵解并大量生成乳酸^[12]^。乳酸在肺癌TME中通过多种机制发挥促瘤作用^[13]^：其一，通过哺乳动物雷帕霉素靶蛋白复合物2（mammalian target of rapamycin complex 2, mTORC2）/AKT通路或H3K18组蛋白乳酸化修饰稳定缺氧诱导因子-1α（hypoxia-inducible factor-1α, HIF-1α）诱导TAMs向M2促瘤表型极化，同时分泌血小板衍生生长因子、血管内皮生长因子（vascular endothelial growth factor, VEGF）等前纤维化和促修复因子，诱导肺泡上皮细胞上皮-间充质转化及成纤维细胞向肌成纤维细胞转化，促进纤维化与组织修复，与肿瘤生长、血管生成、转移及不良预后密切相关^[14]^；其二，直接抑制CD8^+ ^T细胞的p38/c-Jun氨基末端激酶（c-Jun N-terminal kinase, JNK）通路和自然杀伤细胞的细胞毒活性，形成免疫豁免TME。此外，肺癌慢性缺氧的病理特征会进一步调控TAMs糖代谢，使其减少葡萄糖向糖酵解、三羧酸循环的分流，转而通过非经典戊糖磷酸途径高效生成NADPH，在维持细胞稳态、促进胆固醇外排的同时，反向强化胞葬能力^[15]^。目前，精准催化肿瘤内乳酸降解的纳米酶研究已取得初步进展，为靶向干预该代谢环节提供了新方向^[16]^。

### 1.3 肺癌特异性及巨噬细胞异质性

肺癌TME因高度氧化的生理特征及肺泡结构的物理支撑，其胞葬介导的代谢重编程呈现出显著的组织特异性。与肝脏、肠道等实体肿瘤不同，肺癌细胞凋亡后的清除过程还受肺泡表面活性蛋白（surfactant proteins, SPs）调控，在NSCLC中，SPs可作为调理素增强巨噬细胞对凋亡细胞的识别，促进吞噬细胞向M2促瘤表型转化，进一步触发代谢重编程，为异常血管生成及肺间质胶原交联提供始动信号^[17]^；更深层次而言，肺癌TME中TAMs的高度异质性，在很大程度上源于胞葬持续激活后的代谢重编程与转录重塑，驱动巨噬细胞向不同功能亚群分化。如上文所述，持续激活胞葬一方面通过脂质摄取、胆固醇外排及核受体激活，塑造以脂质代谢适应为特征的免疫抑制表型；另一方面通过增强糖酵解、乳酸积累及缺氧适应，推动巨噬细胞向促瘤表型转化，同时获得更强的促血管生成和促基质重塑功能。早期吸烟相关NSCLC患者的单细胞测序研究^[18]^证实，肺癌TME中存在多种免疫抑制性TAMs亚群，且各亚群依赖胞葬持续激活后产生独特的功能代谢谱。如CCL8^+^巨噬细胞因其存在高水平的脂肪酸氧化和氧化磷酸化活性，倾向于通过抑制炎症因子释放发挥免疫抑制作用；而SPP1^+^巨噬细胞则更依赖糖酵解刺激血管生成和基质重塑来协助肿瘤转移。这种TAMs亚群的代谢异质性，是肺癌TME结构重塑及临床表型差异的重要分子基础之一，也提示胞葬可能是驱动不同促瘤巨噬细胞程序分化的重要上游环节。因此，胞葬介导的代谢-免疫级联反应可能成为肺癌间质由生理性顺应向病理性硬化转变的核心诱因，最终构建起具有物理排斥作用的免疫屏障。

## 2 胞葬驱动的肿瘤基质结构重塑：血管异常与纤维化协同演化

肺癌TME中持续激活的胞葬作用诱发的代谢与分泌重编程，成为调控血管生成异常与基质纤维化协同发展的关键环节。与生理性组织修复中短暂、有序的血管生成和基质沉积不同，肺癌TME中胞葬作用会导致血管结构紊乱、基质过度沉积及组织力学特性改变，形成的结构屏障不仅为肿瘤生长、转移提供了病理支撑，更通过限制免疫细胞浸润、降低药物递送效率削弱免疫治疗疗效，成为介导肺癌免疫排斥的重要机制之一（图1）。

**图1:**
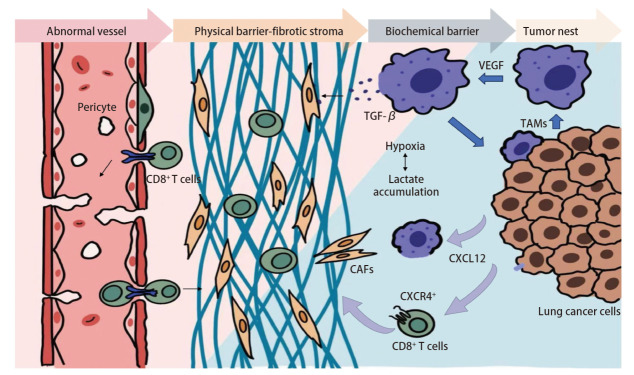
巨噬细胞胞葬驱动肺癌免疫排斥微环境重塑

### 2.1 胞葬诱导的异常血管生成

肺癌的恶性增殖与远处转移依赖新生血管网络的构建，高凋亡负荷下的胞葬作用可通过代谢重编程与分泌谱重塑，最终导致血管过度生成、基底膜不完整及周细胞覆盖率降低，形成高通透性、低灌注效率的异常血管结构，而这也是免疫细胞浸润受限、抗血管生成药物耐药的关键原因之一。

#### 2.1.1 VEGF依赖性机制：代谢-MerTK轴的促血管生成效应

VEGF家族是调控肺癌血管生成的核心分子，而胞葬激活的MerTK信号轴以及下游信号介导的代谢重编程，是肺癌TME中VEGF异常表达的重要诱因。MerTK受体被激活后，可通过PI3K/AKT/mTOR信号通路直接上调VEGF-A的转录与翻译，介导血管内皮细胞的增殖与迁移^[19]^；同时，如上文所述，胞葬诱导的脂质与糖代谢重编程（图2），使TAMs成为TME中VEGF的重要来源，糖酵解导致的乳酸堆积通过HIF-1α依赖方式上调VEGF的表达与分泌，脂质代谢不仅通过HIF-1α依赖方式，而且可通过胞葬后FABP4高表达以及MIF-CD74轴协同激活内皮细胞，形成代谢-血管生成正反馈环路^[20]^，二者协同强化VEGF介导的病理性血管生成。VEGF信号与胞葬过程之间还存在正反馈调控环路，局部高浓度的VEGF可增强TAMs对凋亡细胞的清除能力^[21]^，进一步促进MerTK受体的表达与活化，形成“吞噬-分泌-再吞噬”的恶性循环。

**图2:**
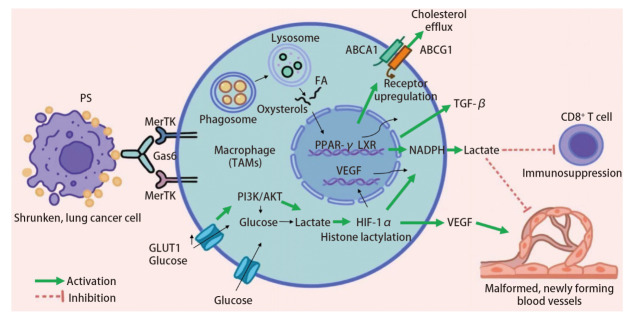
巨噬细胞胞葬驱动的代谢重编程机制图

#### 2.1.2 基质重构与机械信号介导的血管异常

除经典VEGF通路外，TAMs还可通过重塑ECM、调控细胞力学特性，间接促进肺癌血管生成异常。胞葬后发生代谢重编程的TAMs可选择性上调基质金属蛋白酶（matrix metalloproteinases, MMPs）家族成员，通过降解胶原蛋白、纤维连接蛋白等ECM成分，为血管内皮细胞的侵袭与管腔形成创造空间，同时释放ECM中储存的VEGF、血小板衍生生长因子等血管生成因子前体，激活其生物学活性，实现VEGF与非VEGF途径的协同促血管生成效应^[22]^，其中有研究^[23]^指出，MMP-14在NSCLC中高表达与患者不良预后密切相关，是介导肺癌血管重塑的重要分子之一，是精准诊断和治疗的潜在靶点，可能改善NSCLC的整体生存率。

此外，高效胞葬可激活TAMs内的精氨酸酶1通路，导致腐胺等多胺类代谢产物大量积累^[24]^，这类产物可通过调节细胞骨架重排，改变肿瘤细胞与内皮细胞的形态学特征，为血管异常增生提供结构基础。胞葬直接激活的MerTK受体还能磷酸化YAP/TAZ分子，作为调控细胞增殖的核心介质，进一步促进肺癌血管生成与血管拟态形成^[25]^。研究^[26]^表明，经过代谢重编程、*ADAM12*缺失的间充质干细胞通过过度表达*Gas6*、*Lgals3*和*Csf1*等基因，可以增强巨噬细胞细胞作用和M2极化，这一过程可诱导病理性血管生成，进一步加深TME的免疫抑制状态。肺腺癌泛癌分析^[27]^证实，胞葬作用与血管生成呈显著相关，高水平胞葬患者肿瘤血管拟态发生风险更高，且胞葬核心基因*HAVCR1*高表达可通过促进肿瘤细胞增殖、迁移，间接推动血管异常增生，为胞葬介导的肺癌血管生成异常提供了潜在的靶向分子线索。

### 2.2 胞葬介导的基质纤维化与力学TME改变

肺癌TME具有高度纤维化的病理特征，而胞葬作用是驱动该过程的重要诱因之一。TAMs执行胞葬后通过分泌大量促纤维化因子、调控基质胶原交联，将原本疏松的肺间质转化为致密的肿瘤基质，构建起阻挡抗肿瘤免疫细胞浸润的物理-生化双重屏障，这一过程是ICIs治疗失效的重要机制之一。

如上文所述，胞葬激活的PPAR-γ/LXR信号轴可诱导TAMs大量合成并分泌TGF-β，作为诱导纤维化的重要分子之一，胞葬来源的TGF-β一方面以旁分泌形式诱导间质静息成纤维细胞向CAFs表型转化，增强肿瘤细胞的迁徙与侵袭能力^[28]^；另一方面直接抑制树突状细胞的成熟，削弱其抗原呈递能力，阻断抗肿瘤免疫应答的启动^[29]^。有研究^[30]^指出，TGF-β阻断抗体与程序性死亡配体-1（programmed death-ligand 1, PD-L1）联合使用，可降低间质细胞中的TGF-β信号，从而增强T细胞浸润并触发强效抗肿瘤免疫反应。同时，胞葬后的TAMs还高表达赖氨酰氧化酶（lysyl oxidase, LOX），该酶可催化胶原纤维发生共价交联，使松散的胶原网络形成致密、高度排列的纤维结构，可导致肿瘤组织硬度显著增加，促进致密胶原网络形成，并可能参与肿瘤物理性基质屏障的建立^[31]^。

基质纤维化引发的肿瘤力学TME改变，会进一步加剧免疫排斥与肿瘤耐药。基质纤维化一方面通过激活癌细胞及免疫细胞表面的整合素受体，触发FAK/Src信号通路增强肿瘤细胞的抗凋亡能力^[32]^，另一方面通过机械敏感性离子通道Piezo1感知机械应力，诱导CD8^+ ^T细胞功能耗竭^[33]^。有研究^[34]^指出，整合素α3亚基的敲除可以有效减少癌细胞的侵入。同时，基质纤维化能够正反馈促进TAMs向M2型极化^[35]^，进一步分泌促纤维化因子，形成“基质纤维化-免疫抑制-纤维化加剧”的恶性循环，此外，肿瘤基质纤维化仍可通过增强有氧糖酵解^[36]^、上调多重耐药蛋白1的表达，促进肿瘤转移与化疗耐药^[37]^。

### 2.3 血管异常与纤维化的协同演化构建免疫排斥

TME 肺癌TME中，胞葬诱导的血管异常与基质纤维化并非孤立存在，而是相互调控、协同演化，共同促进免疫排斥TME的形成和维持。二者的协同作用通过多重机制强化免疫排斥：一方面，血管高通透性与基质致密化共同阻碍效应T细胞从血管向肿瘤巢的浸润，降低免疫治疗药物的递送效率；另一方面，血管异常与纤维化诱导的TME缺氧、乳酸堆积，可直接抑制CD8^+ ^T细胞与自然杀伤细胞的功能，同时招募调节性T细胞、髓源性抑制细胞等免疫抑制细胞浸润^[38]^。此外，胞葬后TAMs与CAFs协同分泌CXCL12、CXCL8等趋化因子^[39]^，CXCL12可通过其受体CXCR4将效应T细胞诱导向远离肿瘤巢的基质陷阱中，同时特异性招募免疫抑制细胞在屏障边缘集聚，形成生化屏障^[40]^。物理屏障与生化屏障的双重作用，促使肺癌TME从“炎性热肿瘤”向“纤维化冷肿瘤”演变，这也是免疫抑制剂临床应答率受限的重要生物学基础之一。

综上所述，胞葬活性升高不仅参与肺癌免疫排斥型TME的形成，还与ICIs疗效下降存在相关性。一方面，持续胞葬驱动的TAMs免疫抑制化、血管异常及基质致密化，可限制效应T细胞向肿瘤巢核心浸润，使肿瘤呈现典型的“免疫排斥”表型；另一方面，胞葬相关分子上调常伴随MerTK/AXL活化、乳酸代谢增强、TAMs富集及TGF-β信号增强，这些变化均与免疫治疗低应答状态具有一致的生物学方向性。因此，胞葬活性本身及其相关分子网络，可能成为评估肺癌免疫治疗敏感性的重要参考维度之一。

## 3 临床转化：靶向胞葬驱动TME重塑的治疗潜力

随着巨噬细胞胞葬调控肺癌TME结构重塑及免疫排斥的分子机制逐步阐明，靶向胞葬相关信号通路逆转TME病理重构，已成为ICIs原发性或继发性耐药、提升肺癌免疫治疗疗效的重要研究方向。基于胞葬介导的“信号激活-代谢重编程-结构屏障形成-免疫排斥”级联病理反应，临床干预策略可从阻断胞葬信号启动、纠正巨噬细胞功能偏向、破解肿瘤结构屏障三个核心维度展开。

### 3.1 胞葬相关指标作为免疫治疗疗效预测标志物的潜力

在精准免疫治疗时代，单纯依赖PD-L1表达或肿瘤突变负荷（tumor mutational burden, TMB）已难以解释复杂的TME异质性。胞葬作用作为TME的源头，其相关分子的动态变化正成为预测PD-L1抑制剂疗效的关键生物标志物。

从机制上看，如上文所述，若肿瘤组织中存在胞葬受体或桥接分子高表达，或伴随TAMs富集、TGF-β信号增强、乳酸代谢活跃、血管异常和胶原沉积增加，则往往提示TME更偏向免疫抑制和免疫排斥状态，此类患者接受单纯ICIs治疗时获益可能相对有限。

近年来已有多项研究^[41,42]^提出胞葬相关信号用于预测临床结局和免疫治疗反应，提出胞葬相关多基因模型具有预测ICIs效果的潜力。一项泛癌研究^[43]^建立了胞葬多基因评分（efferocytosis potential index, EPI），发现高EPI在多种肿瘤中与免疫浸润和TME特征有关，该研究提出接受免疫检查点治疗的患者中，高EPI可能预测更差的预后。因此，未来可考虑将胞葬相关指标与现有免疫治疗标志物联合应用，构建更具解释力的肺癌特异性疗效预测模型。总体而言，胞葬相关指标不仅具有机制研究价值，也可能成为肺癌精准免疫治疗中兼具预测和分层意义的新型参考标志物。

### 3.2 靶向阻断胞葬信号启动

MerTK与AXL作为TAMs受体酪氨酸激酶家族的核心成员，是介导胞葬信号识别与转导的关键分子，二者在NSCLC中高表达且功能冗余，共同驱动肺癌TME重塑过程以及免疫治疗耐药。针对MerTK/AXL的靶向阻断，可直接抑制TAMs对凋亡细胞的特异性识别与吞噬，从源头阻断胞葬信号的激活；同时可有效抑制TAMs向M2型促瘤表型极化，下调肿瘤细胞及PD-L1的表达水平，有望减轻对效应T细胞的免疫抑制，并部分恢复机体有效的抗肿瘤免疫应答^[44,45]^。针对上游桥接分子Gas6的中和抗体也展现出抗肿瘤潜力^[46]^，血浆可溶性MerTK、Gas6可作为无创监测指标，MerTK/AXL高表达合并表皮生长因子受体（epidermal growth factor receptor, *EGFR*）突变等标志物组合，可实现患者精准分层。

### 3.3 纠正胞葬后功能偏向

相较于完全阻断胞葬的细胞清除功能，纠正胞葬后TAMs的代谢重编程异常及分泌谱偏向，使其恢复“炎症消退且不抑制抗肿瘤免疫”的生理功能表型，更契合TME“正常化”的治疗理念，并尽可能减少凋亡细胞堆积及炎症失控等潜在风险。一方面，靶向胞葬激活的PPAR-γ/LXR/Abca1脂质代谢轴，可有效逆转TAMs的胞内脂质积累，抑制促瘤因子的合成与分泌^[47]^；另一方面，靶向胞葬后异常增强的糖代谢，可减少乳酸堆积，解除乳酸介导的免疫抑制。此外，特异性促消退介质（specialized pro-resolving mediators, SPMs）^[48]^可诱导TAMs向生理性炎症消退表型转化，重塑正常血管结构、减轻基质硬化，恢复TME对免疫细胞的渗透性。

### 3.4 破解结构重构屏障

胞葬作用介导的血管生成异常与基质纤维化重构，是肺癌免疫排斥性结构屏障形成的核心病理基础，针对该病理环节实施抗血管生成与抗纤维化的联合干预，并与ICIs协同应用，是突破肺癌免疫治疗疗效瓶颈的直接且关键的临床策略。靶向抗血管生成可通过靶向抑制VEGF信号通路，实现肿瘤血管的结构与功能正常化重塑，有效缓解肿瘤局部间质液压升高与慢性缺氧的病理状态，不仅为效应T细胞的肿瘤浸润拓展了空间，同时显著提升免疫治疗药物的肿瘤组织递送效率。靶向TGF-β信号通路的阻断剂^[49]^及尼达尼布^[50]^等抗纤维化药物，可特异性抑制CAFs的活化增殖与胶原纤维的共价交联，有效降低肿瘤基质的力学硬度，破坏胞葬驱动形成的致密胶原物理屏障，促进效应T细胞穿透基质屏障抵达肿瘤巢核心发挥杀伤功能。

有研究^[51]^证实PD-L1/VEGF双特异性抗体在肺癌一线治疗中的显著获益，为靶向胞葬相关血管生成通路与免疫检查点的联合干预提供了新型药物形式，未来可进一步探索该类双抗与胞葬信号通路抑制剂（如MerTK/AXL抑制剂）的联合应用，为肺癌免疫治疗耐药提供更精准的解决方案。

## 4 总结与展望

胞葬已不再被视为单纯的细胞残骸清除过程，而是肺癌TME演进中连接细胞死亡、代谢重编程、血管异常、基质纤维化以及免疫抑制的关键枢纽。然而，现有证据虽然提示胞葬与肺癌TME重塑密切相关，但其在不同病理类型、不同分期以及不同治疗背景下，仍缺乏足够的肺癌特异性证据支持。尽管靶向MerTK等受体及相关代谢通路的临床转化研究已初见端倪，但要实现肺癌患者的精准获益，需要识别肺癌病理类型特异性以及空间异质性的差异，动态监测胞葬活性以及设计针对胞葬干预以及炎症稳态的最佳策略。未来应结合单细胞测序、空间转录组及多重免疫荧光等技术，解析高胞葬活性巨噬细胞亚群的空间分布与功能特征，并探索胞葬相关标志物作为免疫治疗分层标志物的潜力。推动抗胞葬、抗血管生成、抗纤维化与免疫治疗的精准联合，有望为突破肺癌免疫治疗瓶颈提供新的研究方向。另外，随着曲妥珠单抗（Trastuzumab deruxtecan, T-DXd）等抗体偶联药物（antibody-drug conjugates, ADCs）在NSCLC中的广泛应用^[52]^，临床观察到了前所未有的肿瘤溶解效应，我们有理由认为ADCs诱发的肺毒性^[53]^不仅源于肿瘤负荷对肺泡上皮的直接损伤，持续激活的胞葬作用同样成为肺间质损伤的重要来源之一。因此，通过精准调节胞葬速率有望成为ADCs伴随治疗的新范式。
